# Burnout syndrome among medical nurse-technicians in intensive care units in cardiovascular surgery

**DOI:** 10.3389/fpubh.2023.1287756

**Published:** 2023-11-17

**Authors:** Aleksandar Nenadovic, Snezana Radovanovic, Stefan Joksimovic, Jagoda Gavrilovic, Marija Sorak, Marko Spasic, Nela Djonovic, Dragan Vasiljevic, Dalibor Stajic, Gordana Djordjevic, Ognjen Djordjevic, Jelena Vuckovic-Filipovic, Radica Zivkovic Zaric, Marija Sekulic

**Affiliations:** ^1^Clinic for Anesthesia and Intensive Care, Institute for Cardiovascular Diseases Dedinje, Belgrade, Serbia; ^2^Department of Social Medicine, Faculty of Medical Sciences, University of Kragujevac, Kragujevac, Serbia; ^3^Center for Harm Reduction of Biological and Chemical Hazards, Faculty of Medical Sciences, University of Kragujevac, Kragujevac, Serbia; ^4^Surgical Oncology Clinic, Institute for Oncology and Radiology of Serbia, Belgrade, Serbia; ^5^Department of Infectious Diseases, Faculty of Medical Sciences, University of Kragujevac, Kragujevac, Serbia; ^6^Department of Gynecology and Obstetrics, Faculty of Medical Sciences, University of Kragujevac, Kragujevac, Serbia; ^7^Department of Surgery, Faculty of Medical Sciences, University of Kragujevac, Kragujevac, Serbia; ^8^Department of Hygiene and Ecology, Faculty of Medical Sciences, University of Kragujevac, Kragujevac, Serbia; ^9^Department of Epidemiology, Faculty of Medical Sciences, University of Kragujevac, Kragujevac, Serbia; ^10^Department of Internal Medicine, Faculty of Medical Sciences, University of Kragujevac, Kragujevac, Serbia; ^11^Department of Pharmacology and Toxicology, Faculty of Medical Sciences, University of Kragujevac, Kragujevac, Serbia

**Keywords:** burnout syndrome, medical nurse-technicians, intensive care units, cardiovascular surgery, Serbia

## Abstract

**Background:**

Healthcare workers in intensive care units work under specifically hard conditions compared to healthcare workers who work under regular clinical conditions. In this sense, the research aims at assessing the level of burnout symptomatology among nurse technicians working in intensive care units for cardiovascular surgery and to compare those burnout levels with those recorded for medical technicians working under regular clinical conditions.

**Method:**

The research was designed as a cross-sectional study. The sample consisted of nurse technicians working in intensive care units specializing in cardiovascular surgery (70 participants) and nurse technicians working under regular clinical conditions (70 participants) at the Institute for Cardiovascular Diseases “Dedinje,” Belgrade, Serbia. To evaluate the manifestation of burnout syndrome at work, the analysis uses the Serbian version of the Maslach Burnout Inventory-Human Services Survey (MBI-HSS).

**Results:**

To examine the variances in the average sub-scores for burnout within two groups of medical technicians, the study used Two Independent Samples *T*-test. The statistically noteworthy differentiation was ascertained for emotional exhaustion and personal accomplishment, but this does not hold true for depersonalization. However, the mean score values across the different burnout levels (low, moderate, high) are similar in two cohorts of respondents (*p* > 0.05).

**Conclusion:**

This study will serve as an impetus for a policy reform focused on ameliorating working conditions and improving healthcare workers' satisfaction and overall healthcare quality.

## Introduction

The work-related burnout syndrome is a condition characterized by a state of protracted involvement in emotionally demanding circumstances, which results in a tripartite manifestation of physical, emotional, and cognitive exhaustion. The operational characterization stratifies burnout into three primary domains: emotional fatigue, depersonalization, and personal accomplishment. Freudenberger and Maslach are forbearers in elucidating the intricacies of occupational burnout (1, 2). The World Health Organization (WHO) has formally recognized burnout as a “professional phenomenon” within the ambit of the International Classification of Diseases 11th Revision, thus acknowledging its gravity as a pertinent health concern (3). The chronicity of burnout syndrome can lead to deep emotional reactions that, without intervention or guidance aimed at its prevention, can cause damage to the physical and psychological integrity of professionals (4). The evidence suggests that burnout symptoms among healthcare workers are elevated across all specialties and in all countries. However, significant disparities have been observed among geographic regions, specialties, and burnout measurement methodologies. It has been ascertained that registered nurses experience a higher level of burnout compared to other healthcare professionals due to the nature of their profession (5). Nursing is considered to be a high-risk profession due to daily exposure to difficult situations such as death and pain care. In other words, intensive care units (ICU) can be stressful due to high levels of mortality, critical medical conditions, and ethical dilemmas (6). Intensive care units (ICU) constitute a hurried, stressful, intricate environment wherein healthcare professionals routinely provide an array of advanced life support and life-sustaining measures to a diverse spectrum of critically ill patients (7). Healthcare workers in intensive care units work under specifically hard conditions compared to healthcare workers who work under regular clinical conditions. Frontline experts in the ICU are directly engaged in patient care and can be subjected to significant psychosocial stressors, moral distress, and burnout. Burnout can also cause deterioration in the quality of care, increasing the risk of morbidity and mortality in patients due to poor performance and increased possibility of making errors (8).

In this sense, it is interesting to carry out a study on burnout levels in ICUs. So far, no extensive research has been conducted on this topic in Serbia. The aim of this research is to assess the level of burnout symptomatology among nurse technicians working in intensive care units for cardiovascular surgery because they are expected to have higher burnout levels compared to those working in regular clinical settings. Additionally, the study seeks to determine possible correlations between the demographic and socio-economic characteristics of the participants and the degree of burnout experienced in both groups under investigation because it has been reported that the susceptibility to burnout syndrome in ICU nurses is influenced by socio-demographic determinants (youth, unmarried status and limited professional experience within intensive care) and employment-related circumstances (excessive workload and prolonged work duration) (9–12).

## Materials and methods

### Study design

The cross-sectional survey was conducted during March and April 2023.

### Study population

The medical technicians in intensive care units and medical technicians in regular clinical institutions at the cardiovascular surgery department of the Institute for Cardiovascular Diseases “Dedinje,” Belgrade, Serbia who met the inclusion criteria were considered eligible to participate in the study.

### Sampling process

The precise number of respondents within each group was calculated based on the data from the studies with similar study design (7) using the statistical program G^*^ Power 3.1 software for random samples. With an effect size of 0.3, an alpha error of 0.05, and a study power of 0.95, the software computed that a sample size of at least 68 participants would be adequate. The sample size was approximated to 70 respondents, adhering to the study's design involving two cohorts of participants. The 140 subjects were randomly drawn from the total pool of nurses. Anonymous self-reported questionnaires were distributed by department heads to 140 nurse technicians (70 nurse technicians working in intensive care units and 70 nurse technicians working in regular clinical settings).

The inclusion criteria were as follows: the age of 19 years and above, nurse technicians employed on fixed-term or indefinite contracts during the research period.

The exclusion criteria were: younger than 19 years, interns/volunteers, employees on sick leave or annual leave, employees with work discontinuity exceeding 1 year (due to absences for professional development in other institutions, extended absence from the workplace due to illness), employees exposed to significant psychophysical trauma in the preceding 6 months (independent of the professional environment).

All respondents were informed in writing that their participation was voluntary and that the information they provided would be treated as confidential. Participants signed written informed consent to participate in this study.

The research protocol gained ethical approval from the Institutional Ethics Committee. The research adhered rigorously to the guidelines delineated in the Helsinki Declaration and the principles of Good Clinical Practice.

### Research instruments

For assessing socio-demographic variables, a distinct questionnaire was devised for the purposes of this study. The questionnaire comprised inquiries pertaining to socio-demographic information, including gender, age, educational attainment, marital status, household size, number of children, cigarette and alcohol consumption, physical activity, dietary habits, and participants' socioeconomic status. Within the context of work conditions, the questionnaire covered the type of occupation (nurse technician, senior nurse technician), years of work experience, current employment status (permanent, fixed-term), work environment (intensive care or regular clinical conditions, shift work, contact with COVID-19 positive patients, duration of work in the COVID-19 system, contact with contaminated materials during work, availability of protective equipment, and sufficient number of healthcare workers).

To evaluate the manifestation of burnout syndrome at work, we used the translated and culturally-adapted Serbian version of Maslach Burnout Inventory-Human Services Survey (MBI-HSS), (Cronbach's α = 0.72) (13). This instrument assesses three dimensions: (1) emotional exhaustion (2) depersonalization of persons and (3) personal accomplishment. Each item is scored based on respondents' evaluations, ranging from “never” experiencing particular feelings to “feeling a few times a week.” Accordingly, the score range for emotional exhaustion is 0–54 (a score <19 indicates low burnout, 19–26 signifies moderate burnout, and >26 reflects high burnout). The depersonalization score range is 0–30 (<6 signifies low burnout, 6–9 represents moderate burnout, and >9 indicates high burnout). The personal accomplishment evaluation yields scores within the range of 0–48 points (>39 indicates low burnout, 34–39 signifies moderate burnout, and <34 reflects high burnout). High scores in emotional exhaustion and depersonalization correspond to higher levels of burnout, whereas a high score in personal accomplishment corresponds to a lower level of burnout in that dimension (13). The rate of item response was very high (100%) and it demonstrates excellent item-response frequency on the MBI-HSS among Serbian nurse technicians.

### Statistical data analysis

The statistical processing and examination of data were performed with IBM SPSS Statistics 22 software package. Kolmogorov-Smirnov test was applied to test normal distribution. The continuous variables were represented as mean values ± standard deviation, whereas categorical variables were portrayed as the ratio of participants displaying a specific outcome. The chi-square test (χ2) was used for analyzing the association in categorical variables, while the differences in continuous variables were tested by using the independent samples *T* test (in the case of normal distribution of variables), respectively Mann-Whitney test (in the case of non-normal distribution of variables). A multivariable regression model was used to establish the relationship between the dependent variable (burnout syndrome) and the independent variables (sociodemographic characteristics), separately for the group of medical technicians working in intensive care units and medical technicians working in regular clinical conditions. The results were conveyed textually, through tabulation and graphically.

## Results

### Socio-demographic profile of the study cohort

A comprehensive overview of the socio-demographic characteristics of the study cohort is presented in Table 1.

**Table 1:** Socio-demographic characteristics of the study population evaluation of the degree of burnout symptoms in the workplace.

		**Medical technicians**		***p***
		**Intensive care unit** ***n*** = **70**	**Regular clinical conditions** ***n*** = **70**	
Gender	Male	12 (17.1%)	15 (21.4%)	*p =* 0.669
	Female	58 (82.9%)	55 (78.6%)	
Age/X ± SD		36.35 ± 10.01	38.14 ± 12.87	*p =* 0.361
Education/school	Secondary school	31 (44.3%)	31 (44.3%)	*p =* 0.276
	College	19 (27.1%)	26 (37.1%)	
	High school	20 (28.6%)	13 (18.6%)	
Marital status	Single	30 (42.9%)	34 (48.6%)	*p =* 0.527
	Married	37 (52.9%)	31 (44.3%)	
	Divorced	3 (4.3%)	5 (7.1%)	
Number of household members/X ± SD		3.46 ± 1.40	3.23 ± 1.65	*p =* 0.379
Number of children/X ± SD		1.0 ± 1.05	1.01 ± 1.12	*p =* 0.938
Cigarette consumption	Yes	36 (51.4%)	23 (32.9%)	***p** **=*** **0.040**
	No	34 (48.6%)	47 (67.1%)	
Alcohol consumption	Yes	16 (22.9%)	14 (20%)	*p =* 0.837
	No	54 (77.1%)	56 (80%)	
Nutrition	Regular	24 (34.3%)	15 (21.4%)	*p =* 0.131
	Irregular	46 (65.7%)	55 (78.6%)	
Physical activity	Yes	36 (51.4%)	34 (48.6%)	*p =* 0.866
	No	34 (48.6%)	36 (51.4%)	
Material status	Very good	3 (4.3%)	4 (5.7%)	*p =* 0.067
	Good	64 (91.4%)	55 (78.6%)	
	Bad	3 (4.3%)	11 (15.7%)	
Work experience/year/X ± SD		15.0 ± 9.52	16.45 ± 12.54	*p =* 0.440
Employment Status	Unspecified	57 (81.4%)	57 (81.4%)	*p =* 1
	Certain	13 (18.6%)	13 (18.6%)	
Organization of work	Shift work	64 (91.4%)	28 (40%)	***p*** **<** **0.001**
	Shift work + on-call	15 (21.4%)		
	One shift	6 (8.6%)	26 (37.1%)	
	Other	/	1 (1,4%)	
Contact with persons with COVID-19 infection	Yes	60 (85.7%)	64 (91.4%)	*p =* 0.426
	No	10 (14.3%)	6 (8.6%)	
Use of protective equipment	Yes	70 (100%)	70 (100%)	/
	No	/	/	

The bold values are statistical significance.

The average score for emotional exhaustion among medical technicians employed in intensive care units was 22.47 ± 10.19 (min 1, max 54), whereas it was 21.43 ± 11.94 (min 0, max 45) within the group of medical technicians working under standard clinical conditions. Although the mean sub-score for emotional fatigue was marginally higher in the subset of medical technicians stationed in intensive care units when compared to their counterparts in routine clinical settings (implying a greater level of professional burnout among intensive care unit technicians), no statistically significant distinction was recorded between these two participant subsets (Independent samples *T* test, *p* = 0.579).

The average score for depersonalization among medical technicians employed in intensive care units amounted to 4.10 ± 5.05 (min 0, max 18) and it was 3.31 ± 4.61 (min 0, max 24) for medical technicians working in regular clinical conditions. A higher depersonalization sub-score within the group of medical technicians in intensive care units suggests an increased level of burnout. Nevertheless, the disparity in depersonalization between the two cohorts of participants did not yield statistically significant results (Mann Whitney test, *p* = 0.764).

The average value of the score for personal accomplishments among the group of medical technicians engaged in the units of intensive care amounted to 34.74 ± 11.27 (minimum 2, maximum 48), while the same value for the cohort of medical technicians working under regular clinical conditions was 35.23 ± 10.24 (minimum 6, maximum 48) (Mann Whitney test, *p* = 0.965). Lower sub-scores for personal accomplishments within the subset of technicians in intensive care units also supports the assumption that burnout syndrome is more pronounced (although not statistically significant) within this group compared to technicians in standard clinical environments. In alternate terms, no statistically significant distinction was evidenced in the mean sub-scores concerning the manifestation of burnout syndrome between the two groups of respondents.

As observable from Table 2, upon scrutinizing variances in the average sub-scores for the degree of burnout within the context of two distinct assemblies of medical technicians, a statistically noteworthy differentiation was recorded for emotional exhaustion and personal accomplishment with Two Independent Samples *T*-test. However, this did not hold true for depersonalization. On the other hand, the mean score values across the different burnout levels (low, moderate, high) were similar for the two cohorts of respondents (*p* > 0.005).

**Table 2:** Relationship between mean subscale scores and the extent of “burnout” across two participant groups.

**MBI-HSS subscores**		**Medical technicians**					

		**Intensive care unit**		**Regular clinical conditions**		**P** ^*^	**P** ^**^
		***n*** **(%)**	**X** ±**SD**	***n*** **(%)**	**X** ±**SD**		
Emotional exhaustion	Low “burn” < 19	30 (42.9%)	13.20 ± 4.12	28 (40.0%)	9.07 ± 5.71	0,793	**0.005**
	Moderate “burn” 19–26	15 (21.4%)	22.80 ± 2.43	15 (21.4%)	23.20 ± 2.81	1	0.624
	High “burn” >26	25 (35.7%)	33.40 ± 6.54	27 (38.6%)	33.26 ± 5.35	0.782	0.861
Depersonalization	Low “burn” < 6	56 (80%)	1.39 ± 1.56	48 (68.6%)	1.10 ± 1.66	**< 0.001**	0.138
	Moderate “burn” 6–9	7 (10%)	7.43 ± 1.27	9 (12.9%)	7.44 ± 1.33	0.682	0.981
	High “burn” >9	7 (10%)	14.57 ± 4.31	13 (18.6%)	12.84 ± 2.79	0.587	0.393
Personal accomplishment	Low “burn” >39	30 (42.9%)	40.46 ± 2.54	30 (42.9%)	42.83 ± 2.82	0.232	**0.017**
	Moderate “burn” 34–39	14 (20%)	36.62 ± 1.76	8 (11.4%)	37.07 ± 1.43	0.272	0.570
	High “burn” < 34	26 (37.1%)	24.31 ± 8.02	32 (45.7%)	24.21 ± 7.05	0.249	0.748

* Chi-square (χ2) test.

** Independent samples *T* test/Mann Whitney test. The bold values are statistical significance.

Namely, it has been ascertained that medical technicians within intensive care units manifest a moderately heightened degree of emotional exhaustion within the context of the low “burnout” classification in relation to the cohort of medical technicians working in conventional clinical settings (13.20 ± 4.12 vs. 9.07 ± 5.71) (*p* = 0.005). Analogously, a statistically significant divergence has been underscored within the low “burnout” classification for personal accomplishment, signifying that the mean metric was comparatively lower in medical technicians (or rather, a more pronounced “burnout” state) in the intensive care units as opposed to their counterparts engaged in regular clinical environments (40.46 ± 2.54 vs. 42.83 ± 2.82) (*p* = 0.017).

In the subsequent phase of our investigation, we delved deeper into the average subscale scores associated with burnout syndrome, considering the socio-demographic attributes within the cohort of medical technicians in intensive care units (Table 3) and the cohort of medical technicians in conventional clinical environments (Table 4). Drawing upon the collected data, it can be inferred that there is a discrepancy in the intensity of burnout with respect to gender in terms of depersonalization among the technicians in intensive care units. Notably, the higher average score recorded among male individuals serves as an indicator of an amplified levels of burnout (*p* = 0.008). Similarly, our findings demonstrate a positive correlation between physical activity and personal accomplishment in this group. Specifically, our results suggest that a greater degree of burnout within the realm of personal accomplishments is present among technicians who are not engaged in regular physical activity (*p* = 0.022).

**Table 3:** Presentation of the relationship between components of burnout syndrome and the socio-demographic characteristics of participants working in intensive care units.

		**Burnout syndrome**					
		**Emotional exhaustion**	**P** ^*^	**Depersonalization**	**P** ^*^	**Personal accomplishment**	**P** ^*^
Gender	Male	23.58 ± 7.90	0.681	5.25 ± 4.11	**0.008**	31.0 ± 13.34	0.322
	Female	22.24 ± 10.64		2.91 ± 4.64		36.10 ± 9.38	
Age	19–35	20.81 ± 9.75	0.214	3.75 ± 5.09	0.366	35.62 ± 10.24	0.684
	36–64	23.86 ± 10.46		2.94 ± 4.20		34.89 ± 10.36	
Education/school	Secondary school	21.80 ± 12.14	0.626	3.32 ± 4.37	0.981	34.29 ± 11.20	0.861
	College	21.57 ± 9.29		3.16 ± 3.74		35.21 ± 10.91	
	High school	24.35 ± 7.57		3.45 ± 5.80		36.70 ± 8.14	
Marital status	Single	21.30 ± 10.36	0.638	2.50 ± 3.04	0.442	37.26 ± 9.24	0.099
	Married	23.56 ± 10.20		3.89 ± 5.61		34.43 ± 10.11	
	Divorced	20.67 ± 10.21		4.33 ± 3.78		24.66 ± 17.24	
Number of household members	≤ 3	23.17 ± 10.83	0.639	3.32 ± 3.86	0.992	33.53 ± 10.17	0.262
	>3	22.00 ± 9.84		3.31 ± 5.09		36.35 ± 10.25	
Number of children	≤ 2	22.47 ± 10.35	0.987	3.23 ± 4.56	0.589	35.20 ± 9.89	0.934
	>2	22.40 ± 8.48		4.40 ± 5.68		35.60 ± 15.56	
Cigarette consumption	Yes	24.42 ± 9.62	0.101	3.66 ± 5.19	0.597	33.94 ± 10.12	0.284
	No	20.41 ± 10.51		2.94 ± 3.95		36.58 ± 10.34	
Alcohol consumption	Yes	24.18 ± 7.96	0.447	4.12 ± 4.60	0.227	32.81 ± 9.18	0.129
	No	21.96 ± 10.77		3.07 ± 4.63		35.94 ± 10.50	
Nutrition	Regular	21.0 ± 10.36	0.387	2.79 ± 3.86	0.622	34.04 ± 11.09	0.488
	Irregular	23.23 ± 10.13		3.58 ± 4.97		35.84 ± 9.83	
Physical activity	Yes	20.72 ± 8.77	0.141	4.05 ± 5.08	0.102	38.05 ± 6.64	**0.022**
	No	24.32 ± 11.34		2.53 ± 3.97		32.55 ± 12.24	
Material status	Very good	24.67 ± 9.61	0.797	0.33 ± 0.57	0.261	45.0 ± 3.64	0.195
	Good	22.50 ± 10.39		3.42 ± 4.72		34.96 ± 10.33	
	Bad	19.67 ± 8.14		4.0 ± 4.0		31.0 ± 8.18	
Work experience/year	1–20	21.56 ± 9.94	0.498	3.75 ± 5.11	0.459	34.87 ± 10.77	0.793
	21–45	23.23 ± 10.47		2.94 ± 4.17		35.52 ± 9.90	
Employment Status	Unspecified	23.12 ± 10.50	0.266	3.49 ± 4.93	0.793	34.49 ± 10.64	0.210
	Certain	19.61 ± 8.48		2.54 ± 2.82		38.46 ± 7.75	
Organization of work	Shift work	22.42 ± 9.6	0.769	3.57 ± 4.77	0.176	35.00 ± 10.66	0.825
	Shift work + on-call	16.00		2.00		40.00	
	One shift	24.00 ± 16.52		0.83 ± 1.60		36.83 ± 5.38	
	Other	/		/		/	
Contact with persons with COVID-19 infection	Yes	22.03 ± 9.80	0.382	3.02 ± 4.50	0.189	35.65 ± 9.98	0.403
	No	25.10 ± 12.56		5.10 ± 5.11		32.70 ± 11.90	

The bold values are statistical significance. ^*^Independent samples *T* test/Mann Whitney test.

**Table 4:** Presentation of the relationship between components of burnout syndrome from the perspective of socio-demographic attributes of participants engaged in regular clinical conditions.

		**Burnout syndrome**					
		**Emotional exhaustion**	***p***	**Depersonalization**	***p***	**Personal accomplishment**	***p***
Gender	Male	16.93 ± 10.93	0.100	4.33 ± 6.28	0.812	39.26 ± 9.04	0.080
	Female	22.65 ± 12.00		4.03 ± 4.73		33.50 ± 11.57	
Age	19–35	17.88 ± 11.19	**0.015**	3.67 ± 5.16	0.354	36.44 ± 11.52	0.223
	36–64	24.77 ± 11.79		4.50 ± 4.99		33.14 ± 10.94	
Education/ school	Secondary school	22.54 ± 12.12	0.768	4.58 ± 5.30	0.062	33.90 ± 11.74	0.066
	College	20.84 ± 11.91		4.73 ± 5.08		32.53 ± 11.30	
	High school	19.92 ± 12.24		1.69 ± 3.90		41.15 ± 7.96	
Marital status	Single	18.94 ± 11.22	0.120	4.41 ± 5.53	0.656	36.67 ± 11.31	0.370
	Married	24.71 ± 12.56		4.22 ± 4.84		32.71 ± 11.39	
	Divorced	18.00 ± 9.30		1.20 ± 1.30		34.20 ± 10.03	
Number of household members	≤ 3	19.09 ± 11.58	**0.044**	3.85 ± 5.09	0.299	35.47 ± 11.13	0.509
	>3	24.92 ± 11.80		4.46 ± 5.07		33.64 ± 11.60	
Number of children	≤ 2	20.38 ± 11.58	**0.041**	3.95 ± 5.05	0.527	34.80 ± 11.53	0.897
	≤ 2	29.50 ± 12.35		5.25 ± 5.31		34.25 ± 9.67	
Cigarette consumption	Yes	20.69 ± 13.61	0.722	4.17 ± 4.93	0.711	35.69 ± 12,63	0.624
	No	21.78 ± 11.17		4.06 ± 5.16		34.27 ± 10.66	
Alcohol consumption	Yes	20.85 ± 12.90	0.843	6.28 ± 6.55	0.135	33.37 ± 11.82	0.060
	No	21.57 ± 11.80		3.55 ± 4.51		40.21 ± 6.54	
Nutrition	Regular	19.73 ± 12.37	0.539	6.06 ± 6.17	0.174	35.53 ± 9.89	0.762
	Irregular	21.89 ± 11.89		3.56 ± 4.62		34.52 ± 11.69	
Physical activity	Yes	19.08 ± 10.70	0.112	4.47 ± 5.37	0.710	35.32 ± 12.52	0.679
	No	23.63 ± 12.75		3.75 ± 4.78		34.19 ± 10.10	
Material status	Very good	19.75 ± 11.41	0.652	2.75 ± 3.20	0.848	42.25 ± 7.04	0.340
	Good	20.94 ± 11.59		4.40 ± 5.30		33.94 ± 11.54	
	Bad	24.45 ± 14.34		3.09 ± 4.36		36.00 ± 10.74	
Work experience/year	1–20	19.19 ± 12.28	**0.029**	4.50 ± 5.17	0.368	35.02 ± 10.95	0.777
	21–45	25.70 ± 10.17		3.33 ± 4.83		34.21 ± 12.09	
Employment Status	Unspecified	23.05 ± 11.77	**0.016**	4.56 ± 5.31	0.092	33.91 ± 11.09	0.199
	Certain	14.31 ± 10.24		2.07 ± 3.20		38.38 ± 11.78	
Organization of work	Shift work	21.25 ± 12.85	0.163	4.57 ± 5.43	0,260	33.35 ± 11.95	0.763
	Shift work + on-call	16.53 ± 10.80		2.86 ± 5.47		36.13 ± 11.87	
	One shift	23.88 ± 10.97		4.04 ± 4.33		35.69 ± 10.56	
Contact with persons with COVID-19 infection	Yes	21.60 ± 12.25	0.682	4.37 ± 5.18	0.154	34.37 ± 11.48	0.377
	No	19.50 ± 8.38		1.16 ± 1.83		38.66 ± 8.52	

The bold values are statistical significance.

Table 4 testifies to the fact that among medical technicians working under conventional conditions, emotional exhaustion is more pronounced in older individuals (*p* = 0.015), those with a higher number of household members (*p* = 0.044), those with a work experience exceeding 20 years (*p* = 0.029), and those employed on an indefinite basis (*p* = 0.016).

Based on the results of the multivariable regression analysis, we can conclude that the prevalence of burnout syndrome among medical technicians from intensive care units depends on marital status, physical inactivity (in terms of its impact on personal accomplishment). As demonstrated in Table 5, the prevalence of burnout syndrome among medical technicians working in regular clinical conditions depends on the following independent variables: age, number of children, length of service, indefinite employment (in terms of its impact on emotional exhaustion), middle, and higher level of education, alcohol consumption (in terms of influence on personal accomplishment).

**Table 5:** Multivariable regression analysis of the association of socio-demographic characteristics with the burnout syndrome through two participant groups.

**Variables**		**Burnout syndrome intensive care unit**					
		**Emotional exhaustion**		**Depersonalization**		**Personal accomplishment**	
		**OR (95%CI)**	***p***	**OR (95%CI)**	***p***	**OR (95%CI)**	***p***
Gender	Male	1.955 (1.342–2.493)	0.681	2.336 (0.550–5.223)	0.111	0.916 (0.514–1.307)	0.117
	Female	1		1		1	
Age		0.164 (0.081–0.327)	0.510	0.072 (0.039–0.150)	0.484	0.158 (0.087–0.402)	0.202
Education/ school	Secondary school	2.544 (1.424–3.337)	0.391	1.996 (1.127–2.807)	0.925	2.410 (1.511–4.430)	0.419
	College	2.771 (1.340–3.798)	0.403	2.701 (0.863–3.286)	0.846	1.489 (0.810–2.123)	0.654
	High school	1		1		1	
Marital status	Single	0.786 (0.633–1.305)	0.919	1.883 (0.742–3.758)	0.515	1.260 (0.465–2.473)	**0.042**
	Married	0.901 (0.411–1.521)	0.640	1.441 (0.510–2.777)	0.874	0.976 (0.264–1.796)	0.110
	Divorced	1		1		1	
Number of household members		0.991 (0.760–2.512)	0.389	0.687 (0.301–1.276)	0.222	0.996 (0.855–2.612)	0.335
Number of children		1.807 (0.539–2.886)	0.648	0.759 (0.069–1.364)	0.571	1.818 (0.539–2.897)	0.649
Cigarette consumption	Yes	4.005 (0.798–8.808)	0.101	1.486 (0.725–2.937)	0.515	1.644 (0.752–2.237)	0.284
	No	1		1		1	
Alcohol consumption	Yes	3.583 (2.225–8.032)	0.447	1.051 (0.798–2.679)	0.428	0.691 (0.017–1.076)	0.186
	No	1		1		1	
Nutrition	Regular	2.039 (1.239–2.892)	0.387	2.795 (1.532–3.123)	0.498	1.806 (0.971–3.359)	0.488
	Irregular	1		1		1	
Physical activity	Yes	3.601 (1.221–7.423)	0.141	1.096 (0.661–1.526)	0.168	0.764 (0.550–1.024)	**0.024**
	No	1		1		1	
Material status	Very good	2.181 (1.181–5.00)	0.555	2.267 (0.969–3.890)	0.366	1.400 (0.529–3.052)	0.096
	Good	1.498 (0.931–2.833)	0.644	0.578 (0.211–1.045)	0.833	1.069 (0.799–1.592)	0.510
	Bad	1		1		1	
Work experience/year		0.156 (0.102–0.360)	0.431	0.079 (0.038–0.155)	0.520	0.193 (0.116–0.375)	0.375
Employment Status	Unspecified	4.507 (2.773–9.748)	0.266	1.889 (0.953–3.794)	0.506	2.283 (1.022–3.970)	0.210
	Certain	1		1		1	
Organization of work	Shift work	0.722 (0.571–1.104)	0.722	2.738 (1.197–4.673)	0.169	1.067 (0.700–1.833)	0.680
	Shift work + on-call	1.421 (0.800–3.021)	0.475	1.167 (0.984–1.780)	0.816	2.551 (1.917–3.167)	0.778
	One shift	1		1		1	
Contact with persons with COVID-19 infection	No	1.487 (0.879–3.892)	0.382	2.083 (1.044–5.211)	0.188	0.950 (0.045–1.945)	0.403
	Yes	1		1		1	
**Variables**		**Burnout syndrome regular clinical conditions**					
		**Emotional exhaustion**		**Depersonalization**		**Personal accomplishment**	
Gender	Male	3.434 (1.131–5.721)	0.100	1.483 (0.297–2.663)	0.842	1.780 (0.696–3.234)	0.080
	Female	1		1		1	
Age			**0.005**	0.048 (0.005–0.097)	0.968	0.115 (0.095–0.325)	0.278
Education/ school	Secondary school	2.665 (0.658–3.988)	0.513	2.888 (0.406–4.182)	0.085	1.997 (0.498–3.631)	**0.050**
	College	0.923 (0.106–1.260)	0.823	3.038 (1.791–4.457)	0.078	2.308 (1.165–3.733)	**0.025**
	High School	1		1		1	
Marital status	Single	1.186 (0.458–1.800)	0.240	2.426 (1.630–3.212)	0.190	2.476 (1.302–4.264)	0.648
	Married	1.028 (0.941–1.122)	0.868	1.240 (0.846–2.441)	0.219	1.490 (0.935 −2.034)	0.785
	Divorced	1		1		1	
Number of household members		1.277 (0.728–2.468)	0.407	0.371 (0.035–0.776)	0.925	0.827 (0.148–1.503)	0.859
Number of children		3.157 (0.699–5.614)	**0.013**	0.822 (0.217–1.306)	0.692	1.738 (0.687–3.111)	0.574
Cigarette consumption	Yes	3.058 (1.092–5.010)	0.722	1.296 (0.110–2.696)	0.933	2.885 (1.419–4.339)	0.624
	No	1		1		1	
Alcohol consumption	Yes	3.593 (1.884–6.455)	0.843	2.732 (1.839–5.696)	0.070	2.078 (0.839 −3.291)	**0.041**
	No	1		1		1	
Nutrition	Regular	1.617 (0.129–2.158)	0.539	1.452 (0.395–2.503)	0.089	1.006 (0.604–2.255)	0.762
	Irregular	1		1		1	
Physical activity	Yes	2.823 (1.082–4.551)	0.122	1.704 (0.721–3.145)	0.555	1.129 (0.713–2.713)	0.679
	No	1		1		1	
Material status	Very good	4.705 (1.873–7.030)	0.506	2.796 (1.281–4.600)	0.909	2.250 (0.873–4.227)	0.345
	Good	3.509 (0.882–4.428)	0.381	2.051 (1.309–3.670)	0.440	1.947 (1.095–2.778)	0.583
	Bad	1		1		1	
Work experience/year		0.318 (0.100–0.535)	**0.005**	0.090 (0.008–0.105)	0.877	0.120 (0.096–0.312)	0.379
Employment Status	Unspecified	2.469 (1.678–3.541)	**0.016**	2.484 (0.581–5.550)	0.110	2.410 (1.135–4.472)	0.199
	Certain	1		1		1	
Organization of work	Shift work	2.475 (1.234–3.862)	0.222	2.175 (1.670–3.846)	0.216	2.858 (1.786–4.357)	0.647
	Shift work + on-call	3.065 (1.121–4.756)	0.113	2.962 (1.856–5.222)	0.124	3.133 (1.543–5.170)	0.493
	One shift	1		1		1	
Contact with persons with COVID-19 infection	No	2.109 (1.234–3.976)	0.682	3.208 (1.061–5.249)	0.138	2.667 (1.391–4.822)	0,377
	Yes	1		1		1	

The bold values are statistical significance.

## Discussion

Occupational burnout occurs most frequently in professions of public trust that involve helping other people, which is especially emphasized in case of nursing. The previous studies have confirmed that nursing is the most stressful job among the 40 analyzed professions (14) and that nurses experience very high levels of burnout, dissatisfaction, and work-related stress (15). Nurses work with many people, including patients, families, and co-workers, which exposes them to occupational burnout (16). Some studies have revealed that the overall MBI score was the highest in nurses from intensive care units (17). The results of our research show that medical technicians within intensive care units manifest an averagely heightened levels of emotional exhaustion and personal accomplishment within the context of the low “burnout” classification in relation to the cohort of medical technicians working under conventional clinical circumstances. This did not hold true for depersonalization. However, the mean score values across the different burnout levels (low, moderate, high) exhibited similarity amidst the two cohorts of respondents. In addition, the results indicate that there are certain correlations between the demographic and socio-economic characteristics of the participants and the levels of burnout experienced in both investigated groups. In the cohort of medical technicians working in conventional working conditions, emotional exhaustion is more pronounced in older people, those with a larger number of household members, those with work experience over 20 years, and those with indefinite employment. Among technicians working in intensive care units, our results suggest that a higher levels of personal accomplishment burnout is observed in male technicians who do not engage in regular physical activity.

Some studies have revealed that respondents who are older than 35 years were less likely to develop emotional exhaustion and depersonalization. Longer working hours in intensive care units are associated with a reduced sense of personal accomplishment. Among nurses, males have a lower sense of professional accomplishment, and not exercising regularly is associated with more emotional exhaustion and less depersonalization. Working in cardiology intensive care units makes them less likely to have a reduced sense of personal accomplishment (8).

Other authors emphasize that elevated levels of emotional exhaustion (EE) can be linked to personal factors such as being single and having childcare responsibilities, as well as work-related factors such as long working days, poor quality of work life, and insufficient time for self-emperor (18, 19). Shift work and factors related to work patterns have been explored across various studies which reported ambiguous findings concerning the relationship between nocturnal employment and weekly labor hours and burnout. Nocturnal shift labor has been linked to emotional exhaustion, whereas rotational shift employment heightens shift frequency. Extra work hours have yield burnout outcomes. The quantity of working hours has been reported to be connected to burnout, whereas demands associated with on-call duties demonstrate no significant association with any aspect of the Maslach Burnout Inventory. Prolonged shifts extending to 12 h or more can be associated with all components of the Maslach Burnout Inventory, as well as emotional exhaustion, whereas shorter shifts confer a protective shield against burnout. Having more than 8 days of respite per month has been identified as correlated with reduced burnout (20–22).

Our results show that there is a positive correlation between emotional exhaustion and the age of the respondents, the number of children and the length of service in technicians working under regular conditions. There is no statistically significant correlation among nurses in intensive care units. When it comes to work organization (shift work), the results show that there are no statistically significant differences in emotional exhaustion, depersonalization and personal accomplishment in any of the investigated groups.

This study has many limitations, one of which is that the replies are self-reported. Consequently, the responses are subjective and not backed up by clinical evidence. It is also important to note that certain factors, such as the unequal distribution of genders of the participants (more females than males), have also limited the generalizability of the results obtained through this study. Based on our results, we are not able to conclude which work system affects the professional burnout of nurses. There is a lack of more detailed data on the conditions and organization of work and on human resources. The differences in levels of burnout highlight the importance of considering the clinical settings. These results can be the basis for future, more comprehensive and detailed research. It would be good to investigate what coping strategies nurses can use and what measures they propose for improving working conditions and work organization.

The results indicate a need for tailored interventions and support programs specifically designed for nurses working in high-stress settings such as intensive care units. Programs can focus on providing active coping strategies and seeking emotional and instrumental support. It is also necessary to improve the organization of work. The differences in burnout levels highlight the importance of implementing sustainable practices that support the wellbeing of healthcare workers.

## Conclusion

The results indicate that medical technicians in intensive care units exhubit an averagely higher levels of emotional exhaustion and personal accomplishment in the context of a low burnout classification compared to a cohort of medical technicians working in conventional clinical circumstances. Also, the existence of a correlation between certain demographic and socio-economic characteristics of the participants and the levels of burnout experienced in both investigated groups was determined. Investigations of this nature are both significant and necessary to identify burnout-related factors within nursing and to implement effective interventions and strategies for the mitigation and prevention of burnout among healthcare personnel.

## Data availability statement

The original contributions presented in the study are included in the article/supplementary material, further inquiries can be directed to the corresponding authors.

## Ethics statement

The studies involving humans were approved by Ethics Committee of Institute of Cardiovascular Diseases Dedinje. The studies were conducted in accordance with the local legislation and institutional requirements. The participants provided their written informed consent to participate in this study.

## Author contributions

AN: Writing—original draft. SR: Writing—review & editing. SJ: Conceptualization, Writing—review & editing. JG: Data curation, Methodology, Writing—original draft. MSo: Formal analysis, Supervision, Writing—original draft. MSp: Project administration, Writing—original draft. ND: Validation, Visualization, Writing—original draft. DV: Funding acquisition, Writing—review & editing. DS: Methodology, Writing—review & editing. GD: Supervision, Writing—original draft. OD: Data curation, Writing—original draft. JV-F: Software, Writing—review & editing. RZ: Investigation, Writing—review & editing. MSe: Writing—original draft, Writing—review & editing.
